# Books and Magazines

**Published:** 1906-02

**Authors:** 


### Books and Magazines.

THE NEW UNITED STATES PHARMACOPIA makes many changes in the strength of drugs and preparations, reducing some, increasing others as much as double. The law recognizes the *current* U. S. Pharmacopia as the standard. To avoid accidents and damage suits on the one hand, and puzzling lack of results on the other, both the druggist and doctor must follow the *same* standard. As a convenient pocket reminder of these changes, the importance of which must be at once obvious to every physician and pharmacist, Messrs. Lea Brothers & Co., the Medical Publishers, of 706-8-10 Sansom street, Philadelphia, and 111 Fifth avenue, New York, have issued for *free* distribution a carefully prepared leaflet giving an alphabetical list of the important changes. The strength of each preparation listed is given as in both the old and the new U. S. P.

To aid in preventing untoward or negative results in the use of powerful drugs this leaflet will prove handy and valuable.

A postal card request will bring a copy to any physician, druggist, student or nurse.

A TEXT-BOOK ON MODERN MATERIA MEDICA AND THERAPEUTICS. By A. A. Stevens, A. M., M. D., Lecturer on Physical Diagnosis, University of Pennsylvania; Professor of Pathology, Woman's Medical College of Philadelphia. Fourth revised edition, adapted to the new (1905) Pharmacopeia. Octavo of 670 pages. Philadelphia and London: W. B. Saunders & Co., 1905. Cloth, $3.50, net.

The new fourth edition of Dr. Stevens' excellent work on practical therapeutics appears at a most opportune time, close upon the issuance of the eighth decennial revision of the Pharmacopeia to which it has been adapted. Dr. Stevens, by his extensive teaching experience, has acquired a clear, concise diction that adds greatly to his work's pre-eminence. New articles have been added on Sco-

UNQUESTIONABLY

a specific selective influence upon
the entire respiratory tract
is exerted by

GRAY'S Glycerine TONIC Comp.

It overcomes the ever present
element of constitutional depression

THE PURDUE FREDERICK CO.,
298 Broadway, New York polamin, Ethyl Chlorid, Theocin, Veronal, and Radium, besides much new matter to the section on Radiotherapy. The numerous changes in name or strength of various drugs and preparations, as called for by the new Pharmacopeia, have also been made. In fact, it is somewhat difficult to speak of Dr. Stevens' Therapeutics without resorting to the frequent use of superlatives, for of all the good works on this most important of subjects, this book before us is undoubtedly the very best.

---

Nervous and Mental Diseases. By Archibald Church, M. D., Professor of Nervous and Mental Diseases and Medical Jurisprudence in Northwestern University Medical School, Chicago; and Frederick Peterson, M. D., President of the State Commission in Lunacy, New York; Clinical Professor of Neurology and Psychiatry, Columbia University. Fifth edition, revised and enlarged. Octavo volume of 937 pages, with 341 illustrations. Philadelphia and London: W. B. Saunders & Co., 1905. Cloth, $5, net; half Morocco, $6, net.

It is not at all surprising to us that a fifth edition of Church and Peterson's work should be necessary. Indeed, such a success was to be expected from what is undoubtedly the most complete and authoritative volume on nervous and mental diseases today. In preparing this edition Dr. Church has carefully revised his entire section, placing it in accord with the most recent psychiatric advances. In Dr. Peterson's section—Mental Diseases—the Kræpelin classification of insanity has been added to the chapter on classifications for purposes of reference, and new chapters on Manic-Depressive Insanity and on Dementia Præcox included. While the changes throughout have been many, they have been so made as but slightly to increase the size of the work. A number of the illustrations have been replaced by newer and better ones. We can confidently say that this work will maintain the reputation already won.

---

Diseases of the Eye and Refraction, Including Treatment and Surgery. By George M. Gould, A. M., M. D., Editor of *American Medicine*, and Walter L. Pyle, A. M., M. D., Associate Surgeon to Wills Eye Hospital. A section on Retinoscopy is contributed by Dr. James Thorington. Third edition, revised and enlarged; 109 illustration, several in colors. 12mo of 295 pages. Cloth binding at $1; interleaved, $1.25.

In this new, third edition, the authors have corrected, enlarged and revised the text, until now the volume far exceeds the ordinary size and scope of a compend. The whole book has been reset in larger type, and several new illustrations, including five colored plates, have been added. The section on Local Ocular Therapeutics has been increased to include all the recent mydriatics, miotics, local anesthetics, ocular antiseptics, etc. Additional emphasis has been given to points of practical value.

Dose-Book and Manual of Prescription-Writing: With a List of the Official Drugs and Preparations, and the More Important Newer Remedies. By E. Q. Thornton, M. D., Assistant Professor of Materia Medica, Jefferson Medical College, Philadelphia. Third edition, revised and enlarged, adapted to the New (1905) Pharmacopeia. 12mo, 392 pages, illustrated. Philadelphia and London: W. B. Saunders & Co.. 1905. Bound in flexible leather, $2, net.

A glance at the contents of Dr. Thornton's book explains its attainment of a third edition. In addition to the consideration of the official and more important nonofficial preparations intended for internal administration, weights and measures, solubles, and incompatibilities, attention is given to the grammatic construction of prescriptions, illustrated by examples. In revising the text for this edition Dr. Thornton has made it conform with the new (1905) Pharmacopeia, the radical change in strength or name of many chemicals, drugs, and preparations already official, and the admission of many newer remedies necessitating the rewriting of a number of sections. We notice in the appendix an addition of much value—a table showing the change in strength of important preparations, and also a list of average doses for adults in accordance with the new Pharmacopeia. Dr. Thornton's Dose-Book is, as it always has been, accurate and up-to-date.

---

MALFORMATIONS OF THE GENITAL ORGANS OF WOMAN. By Ch. Debierre, Professor of Anatomy in the Medical Faculty at Lille, has been translated by J. Henry C. Simes, M. D., Emeritus Professor of Genito-Urinary and Venereal Diseases in the Philadelphia Polyclinic. 12mo, 182 pages, 85 illustrations. Price, $1.50.

The translation has been made with the consent of the author in order to fill a void in English medical literature. The anatomy, development, and malformations of the genital organs are, in turn, considered. Dr. Debierre in his preface says: "If we have undertaken to write a new history on this subject, it is that besides the great attraction, the great curiosity which belongs to it, there is also a scientific and practical interest of the first order, which we have endeavored to place in evidence upon every page. It will be for the reader to say, if we have accomplished the two-fold end we essayed to reach—to interest, to instruct."

---

GALL STONES AND THEIR SURGICAL TREATMENT. By B. G. A. Moynihan, M. S. (London), F. R. S. S., Senior Assistant Surgeon to Leeds General Infirmary, Leeds, England. Second edition, revised and enlarged. Octavo of 458 pages, beautifully illustrated. Philadelphia and London: W. B. Saunders & Co., 1905. Cloth, $5, net; half Morocco, $6, net.

The first edition of Mr. Moynihan's work on gall stones was completely exhausted in eight months. Mr. Moynihan, by his masterly presentation of operative technic and clear, logical discussions of indications and contraindications, has won an enviable place in contemporary abdominal surgery. In this edition, increased in size by seventy pages, many additional case records have been incorporated and a number of new illustrations added. We note also the addition of a very valuable chapter—Congenital Abnormalities of the Gall Bladder and Bile Ducts. It is evident that the whole text has undergone a careful revision and all recent work along the line of gall stone surgery included. Mr. Moynihan's book still holds first place in its field. The illustrations are very beautiful, especially the nine colored plates.

